# STAT3 signaling in cancer: mechanisms and targeting therapeutic

**DOI:** 10.3389/fimmu.2026.1911338

**Published:** 2026-08-19

**Authors:** Yuxiang Bai, Ziru Zhang, Jun Zhang

**Affiliations:** Department of Pathology and Forensic Medicine, College of Basic Medical Sciences, Dalian Medical University, Dalian, China

**Keywords:** STAT3, cancer, immunosuppression, immunotherapy, targeted therapy

## Abstract

Signal Transducer and Activator of Transcription 3 (STAT3) plays a crucial role in tumor immunity. STAT3 promote angiogenesis, EMT, CD8^+^ T cell activation and exhaustion and CD4^+^ T cells differentiation. Simultaneously, STAT3 aberrant activation inhibits DC function. Moreover, STAT3 inducts immune evasion, making it one of the most promising targets for cancer therapy. Targeting STAT3 represents a promising potential therapeutic strategy, with several STAT3 inhibitors having advanced into clinical trials. In this review, we outline the regulatory role of STAT3 and its mediated signaling pathways in tumor immunity, summarize STAT3-based targeted therapies and combination immunotherapies.

## 1 Introduction

**Figure 1:**
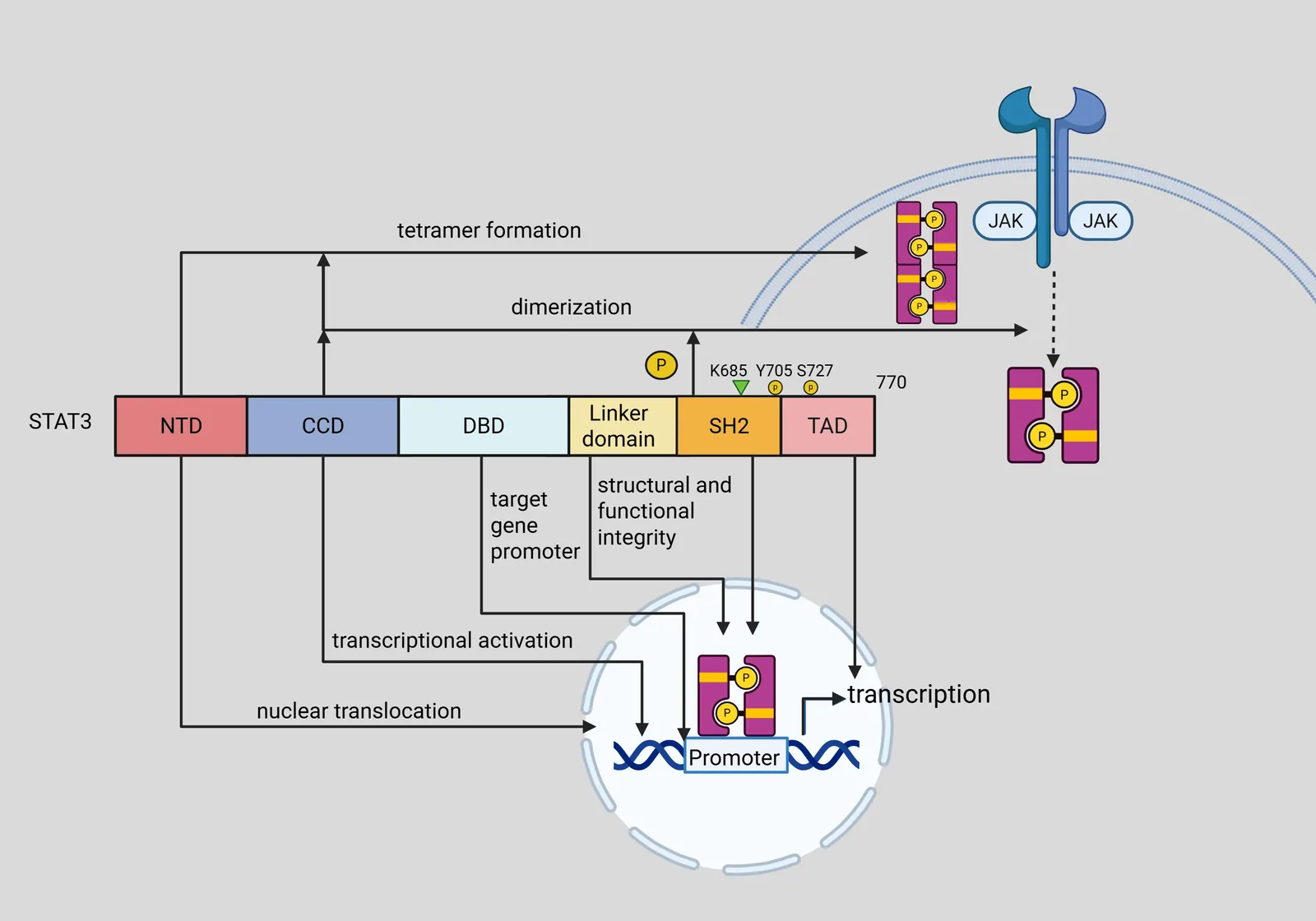
The structure of STAT3. STAT3 is consist of six functionally conserved domains. The STAT3 protein consists of approximately 770 amino acids, with three functionally critical residues, including Lys685, Tyr705, and Ser727, which respectively are located at positions 685, 705, and 727. NTD, N-terminal domain; CCD, the coiled-coil domain; DBD, the DNA-binding domain; SH2, the SRC homology 2 domain; TAD, the carboxyl-terminal transactivation domain. Created with https://www.Biorender.com.

The signal transducer and activator of transcription (STAT) proteins are a family of cytoplasmic transcription factors, comprised STAT1, STAT2, STAT3, STAT4, STAT5a, STAT5b and STAT6. STAT3, one of the most important members of the STAT family, has become a focus for several disease areas as a potential anticancer target, ranging in size from 750 to 850 amino acid residues and characterized by the presence of six functionally conserved domains, including the N-terminal domain (NTD), the coiled-coil domain (CCD), the DNA-binding domain (DBD), the linker domain, the SRC homology 2 (SH2) domain, and the carboxyl-terminal transactivation domain (TAD) (1). Specifically, NTD participates in STAT3 nuclear translocation and tetramer formation. CCD facilities the stabilization of STAT3 dimers and mediates tetramer formation and transcriptional activation. DBD interacts with STAT3 response elements (such as GAS sequences) within target gene promoters. The linker domain ensures the structural and functional integrity of STAT3 by enabling precise inter-domain communication and regulation. SH2 is the most highly conserved domain, which mediates STAT3 binding to phosphorylated tyrosine residues (such as pY705), facilitating dimer formation and nuclear translocation. TAD recruits coactivators (such as p300/CBP) to initiate transcription (2) (**Figure 1**).

Notably, phosphorylation of STAT3 is essential for its activation and this is exerted on C-terminal transactivation domain. Phosphorylation of STAT3 can occur at tyrosine 705 (Tyr705) and serine 727 (Ser727). Upon phosphorylation, STAT3 undergoes a conformational change, enabling dimerization through SH2 domain binding to phosphorylated tyrosine residues. Subsequently, the dimer translocates into the nucleus to promote the transcription activation (3, 4). Beyond phosphorylation, the transcriptional activity of STAT3 is modulated by a spectrum of post-translational modifications, thereby contributing an additional layer of complexity to its persistent aberrant activation in cancer (5). Protein acetylation has generally been revealed to as a crucial post-translational modification (6). STAT3 can be reversibly acetylated on lysine 685 (Lys685), which can enhance DNA methylation and transactivation activity, and promote the phosphorylation on Tyr705 (6, 7). Simultaneously, previously report has discovered that Tyr705-phosphorylation protects Lys685 from deacetylation, forming a positive feedback loop between Tyr705 and Lys685 (8).

Substantial evidence indicates that non-coding RNAs (ncRNAs) can modulate STAT3 activity, either directly or indirectly. Long non-coding RNAs (lncRNAs) and microRNAs (miRNAs), which widely served as various cancer biomarkers, are the most well-studied “classical ncRNA” types (9). STAT3 can upregulate miRNAs expression by directly binding to their promoters. At the same time, specific miRNAs contain conserved STAT3 binding sites, which prevent STAT3 from binding to them, ultimately modulating tumor progression (10). For instance, miR-519a inhibits STAT3 expression by targeting the STAT3/Bcl2 signaling pathway, and thus can act as a tumor suppressor in glioma (11); miR-218 acts as a tumor suppressor in lung cancer via IL-6/STAT3 signaling pathway regulation (12). Additionally, lnc-RNAs, as molecular sponges for miRNAs, regulate STAT3 activity through multiple mechanisms. It not only indirectly regulates the expression of crucial genes in numerous cancers but also directly interacts with miRNAs, leading to degradation or sequestration (13). For example, lncRNA-CCAT5 bound to the C-domain of STAT3 and blocks SH2-containing protein tyrosine phosphatase 1 (SHP-1)-mediated STAT3^Y705^ dephosphorylation, leading to STAT3 nuclear entry and transactivation, thus accelerating gastric cancer progression (14).

## 2 The role of STAT3 in tumor immunity

### 2.1 Promote angiogenesis

**Figure 2:**
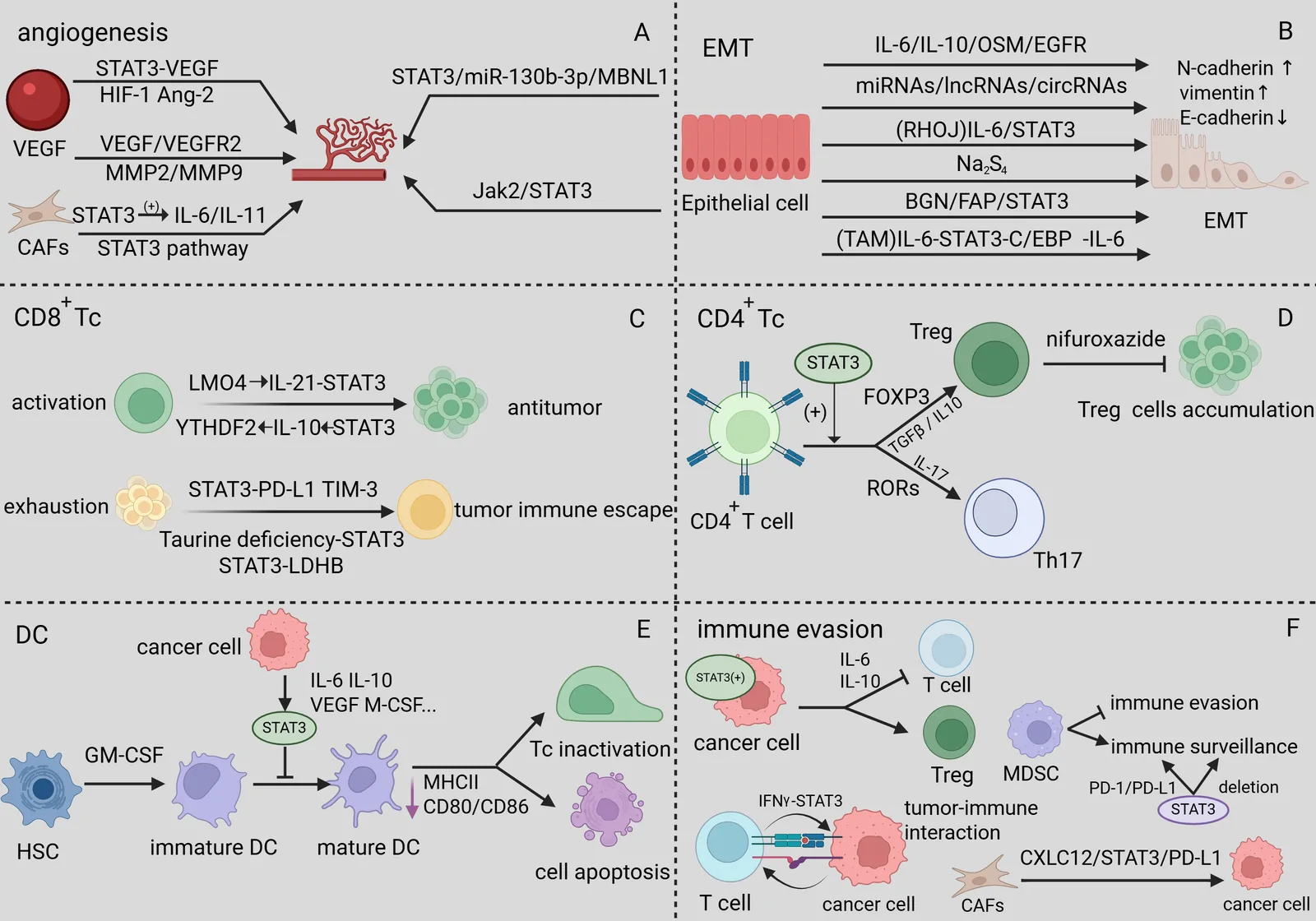
The role of STAT3 in tumor immunity. STAT3 promote angiogenesis **(A)**, EMT **(B)**, CD8^+^ T cell activation and exhaustion **(C)** and CD4^+^ T cells differentiation **(D)**. Simultaneously, STAT3 aberrant activation inhibits DC function **(E)**. Moreover, STAT3 inducts immune evasion **(F)**. VEGF, vascular endothelial growth factor; HIF-1, hypoxia inducible factor-1; Ang-2, angiopoietin-2; MMP2/9, Matrix Metallopeptidase 2/9; CAFs, cancer-associated fibroblasts; EMT, epithelial-mesenchymal transition; OSM, oncostatin (M); EGFR, epidermal growth factor receptor; RHOJ, Ras Homolog Family Member; TAM, tumor-associated macrophage; LMO4, LIM-domain-only 4; TIM3, T-cell Immunoglobulin and Mucin-domain containing molecule 3; LDHB, lactate dehydrogenase (B); Tregs, regulatory T cells; Th17, T helper 17; FOXP3, forkhead box P3; TGFβ, transforming growth factor-β; ROR, RAR-related orphan receptor; DC, dendritic cell; GM-CSF, granulocyte-macrophage colony-stimulating factor; MHC, major histocompatibility complex; Tregs, regulatory T cells; MDSCs, myeloid-derived suppressor cells; IFNγ, non-canonical interferon gamma; PD-1, Programmed death receptor 1; PD-L1, Programmed Death-Ligand. Created with https://www.Biorender.com.

Angiogenesis is a complex and dynamic process regulated by various pro- and anti-angiogenic molecules, which plays an important role in tumor growth, invasion, and metastasis and become a targeted strategy in anti-tumor therapy (15). STAT3, as a central bridge linking angiogenesis and immunosuppression, can directly regulate the expression of proangiogenic factors. Studies have shown that upon activation, STAT3 directly binds to the promoter region of vascular endothelial growth factor (VEGF), and indirectly promotes the expression of hypoxia-inducible factor-1 (HIF-1) and angiopoietin-2 (Ang-2), thereby promoting its transcription and translation (16, 17). In addition to facilitating VEGF/VEGFR2 signaling pathway, STAT3 enhances VEGF expression through basement membrane remodeling, mediating by Matrix Metallopeptidase 2/9 (MMP-2/9) in endothelial cells (18). Hypoxia-induced an ER stress mediator TMTC3 expression potentiates tumor angiogenesis through STAT3/VEGFA pathway in esophageal squamous cell carcinoma (19). Cancer-associated fibroblasts (CAFs), the predominant stromal components in the tumor microenvironment (TME), serve as a pivotal hub for STAT3-dependent angiogenesis and the interaction between tumor cells and microenvironment. CAFs modulate IL-6/STAT3 signaling pathway by upregulating MMP-2/9, and VEGF. Additionally, CAFs can trigger STAT3 signaling pathway independently of VEGF via IL-11, ultimately promote angiogenesis, indicating STAT3 activation as a hallmark of CAF activation (20). Experiments have demonstrated STAT3 activation through IL-6/IL-11 in CAFs facilitates angiogenesis, thus promotes colorectal cancer development (21). CD146^+^CAFs promote angiogenesis and vasculogenic mimicry formation via the STAT3 signaling pathway in endometrial cancer (22). Studies have shown that the STAT3/miR-130b-3p/MBNL1 feedback loop plays a vital role in mammalian target of rapamycin complex 1 (mTORC1) -mediated angiogenesis and tumor progression (23). Studies in glioblastoma multiforme have shown that interferon-induced transmembrane protein 3 causes activation of JAK2/STAT3 signaling, results in enhanced angiogenesis (24) (**Figure 2A**).

### 2.2 Promote epithelial-mesenchymal transition

EMT is a key biological process for tumor cells to acquire migration and invasion abilities, thereby facilitating tumor metastasis. STAT3, as an upstream mediator of EMT, promotes metastasis in brain, thoracic, and gastrointestinal cancers. Cytokines such as IL-6, IL-11and oncostatin M (OSM), as well as growth factors including epidermal growth factor receptor (EGFR) ligands, can induce STAT3 phosphorylation at Tyr705, which enables nuclear translocation via STAT3 homodimerization through the SH2 domain and directly binds to the promoter regions of SNAI1(Snail), SNAI2(Slug), Twist1, zinc finger E-box binding homeobox 1 (ZEB1), and ZEB2, leading to upregulation of N-cadherin and vimentin while suppressing E-cadherin (25). Additionally, different molecular pathways such as miRNAs, lncRNAs and circRNAs modulate STAT3-EMT axis (26). Previous research has shown that lncRNA VCAN-AS1 promotes EMT by activating the STAT3/HIF-1α signaling pathway via competitive inhibition of miR-106a-5p in breast cancer (27). In addition, circKIF4A is upregulated in breast cancer, which functions as a ceRNA for miR-637, promoting the expression of STAT3 and upregulating the expression of N-cadherin and vimentin whereas reducing E-cadherin (28). Study unveiled that Ras Homolog Family Member J (RHOJ) induces EMT through IL-6/STAT3 to aggravate gastric cancer progression (29). In renal tubular epithelial cells, Na_2_S_4_ induced dephosphorylation and deacetylation of STAT3, thereby reducing high glucose-caused EMT progression and other response, providing reference for targets in tumor intervention (30). Studies have shown that mutual interaction between tumor cells and activated mesothelial cells mediated by a BGN/FAP/STAT3 positive feedback loop facilitates peritoneal metastasis of gastric cancer (31). Tumor-associated macrophages (TAMs) promote IL-6 expression by forming an IL6-STAT3-C/EBPβ-IL6 positive feedback loop, inducing the EMT in lung cancer (32) (**Figure 2B**).

### 2.3 The dual impact of STAT3 on CD8^+^ T cell activation and exhaustion

In the anti-tumor protection phase, STAT3 physiologically activates CD8^+^ T cells. Simultaneously, IL-10 and IL-21 induced STAT3 phosphorylation at Tyr705 and Ser727 in CD8^+^ T cells, promoting dual regulation of CD8^+^T cell activation and exhaustion through multiple mechanisms (33). LIM-domain-only 4 (LMO4) enhances CD8^+^ T-cell stemness and tumor rejection by boosting IL-21-STAT3 signaling (34). YTH N6-Methyladenosine RNA Binding Protein F2 (YTHDF2) deficiency in TAMs suppressed tumor growth by reprogramming TAMs, which in turn enhanced CD8^+^T cell-mediated antitumor immunity, while the YTHDF2 expression in TAMs was regulated by IL-10-STAT3 signaling (35).

During the tumor immune escape phase, persistent STAT3 activation driven by chronic antigen stimulation induces CD8^+^ T cell exhaustion by upregulating inhibitory receptors (e.g., PD-L1, TIM-3), while simultaneously disrupting cellular metabolic homeostasis (36, 37). Research has showed that downregulating NF-κB activation factor 1 (Act1) upregulates the expression of PD-L1, and promotes CD8^+^ T cell exhaustion to a PD1^+^Tim3^+^ phenotype by activating STAT3, thereby forming an immunosuppressive microenvironment (38). Taurine deficiency promotes immune evasion and taurine supplementation reinvigorates exhausted CD8^+^ T cells and increases the efficacy of cancer therapies, in a manner dependent on STAT3 activity (39). STAT3 directs Lactate dehydrogenase B (LDHB) expression to limit G6PD activity and mediate disulfidptosis in exhausted CD8^+^T cells, leading to impaired antitumor immunity (40) (**Figure 2C**).

### 2.4 Promoting the differentiation of CD4^+^ T cells into their functional subtypes

In the tumor microenvironment, STAT3 promotes the differentiation of CD4^+^ T cells into immunosuppressive regulatory T (Treg) cells and also facilitates the differentiation of T helper 17 (Th17) cells, thereby impairing the effective antitumor immune response. STAT3 can directly bind to the forkhead box P3 (FOXP3) promoter to induce Treg differentiation, or binding to the promoters of transforming growth factor-β (TGFβ) and IL10 in Tregs. Additionally, IL-10R-mediated STAT3 signaling upregulates cytotoxic T lymphocyte-associated antigen-4 (CTLA-4) on the surface of Tregs, enhancing their immunosuppressive capacity (41). Overexpression of STAT3 stimulates IL-17 production and upregulates RAR-related orphan receptor (RORγt and RORα), accelerating Th17 cell maturation and differentiation (42). Experimental findings demonstrated that STAT3 inhibitor (nifuroxazide) suppresses Treg cells accumulation in the tumor microenvironment, thereby promoting tumor immunity (43) (**Figure 2D**).

### 2.5 Aberrant activation of STAT3 inhibits dendritic cell function

Under normal physiological conditions, hematopoietic stem cells differentiate into immature dendritic cells (DCs) under the action of factors such as granulocyte-macrophage colony-stimulating factor (GM-CSF). DCs are versatile antigen-presenting cells whose migratory capacity is essential for initiating pro-inflammatory as well as tolerogenic immune responses (44). Upon encountering pathogens or danger signals, DCs mature and highly express major histocompatibility complex (MHC) molecules and co-stimulatory molecules (e.g., CD80, CD86) (45). Tumor cells secrete a variety of factors (e.g., IL-6, IL-10, VEGF, M-CSF) that can continuously activate the STAT3 signaling pathway in DCs. Sustained STAT3 activation blocks the normal differentiation and maturation of DCs, resulting in reduced expression levels of MHC class II molecules and co-stimulatory molecules (CD80/CD86) on the DC surface. Such DCs fail to provide effective activation signals to T cells, leading to T cells in activation or even their induction of apoptosis (46). Studies have shown that targeted STAT3 blocking resulted in differentiation of acute myeloid leukemia blasts to antigen presenting cells with an activated DC phenotype, representing a potential immune evasion strategies in acute myeloid leukemia (47) (**Figure 2E**).

### 2.6 Induction of immune evasion

Experimental evidence demonstrates that STAT3-activated cancer stem cells suppress T-cell function through secretion of IL-6 and IL-10, while simultaneously recruiting regulatory T cells (Tregs) and myeloid-derived suppressor cells (MDSCs), thereby evading immune surveillance and promoting immune evasion (48, 49). It is reported that tumor-immune interaction is controlled by non-canonical interferon gamma (IFNγ)-STAT3 signaling and supports tumor immune evasion (50). Moreover, STAT3 regulates PD-L1 transcription, thereby amplifying immune suppressive effects, diminishing the therapeutic efficacy of immune checkpoint inhibitors, and promoting immune evasion (51). Study shown that knockdown of phospholipase A2 group VII (PLA2G7) significantly inhibits STAT3 phosphorylation, leading to PD-L1 suppression and inhibiting immune escape in bladder cancer (52). The high expression of IGFBP2 in high baseline tumor (HTB) stimulated the expression of PD-L1 in M2-TAMs through STAT3 activation, which exerted an immunosuppressive effect (53). Loss of STAT3 inactivates the cyclic adenosine monophosphate–guanosine monophosphate (cGAMP) synthase (cGAS)–STING pathway, leading to reduced type I IFN secretion and an IFN gene signature, which facilitates immune evasion, ultimately promoting metastasis in small cell lung cancer (54). Furthermore, CXCL12, derived from CAFs, activates the JAK2/STAT3 signaling pathway upon binding to its receptor CXCR4, elevating PD-L1 expression and ultimately promoting immune evasion in bladder cancer (55) (**Figure 2F**).

## 3 Targeted therapeutic strategies

**Figure 3:**
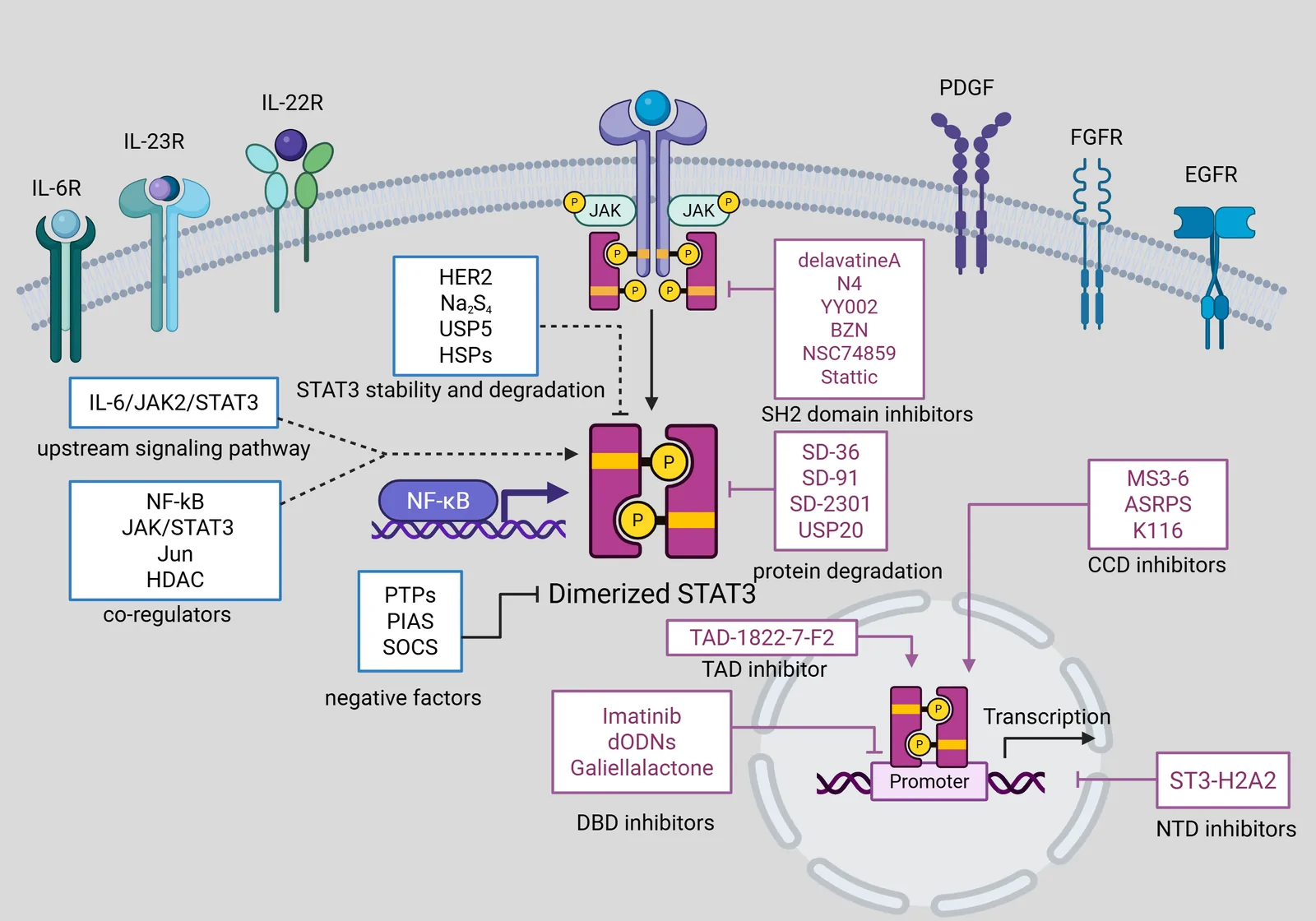
Direct and indirect targeting of STAT3. Direct targeting the STAT3 pathway focuses on the NTD, CCD, DBD, SH2 domain and TAD, also inhibits STAT3-mediated nuclear translocation and induces STAT3 protein degradation. Indirect targeting of STAT3 suppresses its function by inhibiting upstream signaling pathway activation, regulating negative feedback factors, inhibiting co-regulators and impacting on STAT3 degradation. The purple box and solid line represent the direct targeting of STAT3. The blue box and dashed line represent the indirect targeting of STAT3. dODNs, decoy oligodeoxynucleotides; BZN, benzethonium chloride; PTPs, protein tyrosine phosphatases; PIAS, protein inhibitors of activated STATs; SOCS, suppressors of cytokine signaling; USP20, ubiquitin-specific protease 20; PDGF, platelet-derived growth factor; FGF, fibroblast growth factor; EGF, epidermal growth factor; HDAC, histone deacetylases; HSPs, heat shock proteins. Created with https://www.Biorender.com.

STAT3 is extensively activated within the tumor ecosystem, crucially suppressing immune-activating regulators while promoting immunosuppressive factors. Consequently, targeting the STAT3 signaling pathway is a promising therapeutic strategy for multiple cancers (**Table 1**; **Figure 3**).

**Table 1:** Direct and indirect targeting of STAT3.

Type	Molecule	Reference
Direct targeting		
NTD	ST3-H2A2	(59)
CCD	MS3-6	(64)
	ASRPS	(65)
	K116	(63)
DBD	imatinib	(68)
	dODNs	(69)
	Galiellalactone	(70, 71)
SH2	Delavatine A	(73)
	N4	(74)
	YY002	(75)
	BZN	(76)
	NSC74859	(77, 78)
	Stattic	(79)
TAD	TAD-1822-7-F2	(83)
Negative feedback factors	PTPs	(84–86)
	PIAS	(87)
	SOCS	(88, 89)
Protein degradation	PROTAC (SD-36 SD-91 SD-2301)	(90–93)
	USP20	(95)
Indirect targeting		
Upstream signaling pathway	IL-6R IL-23R IL-22R PDGF FGF EGF	(96, 97)
	IL-6/JAK2/STAT3	(98)
Co-regulators	NF-κB	(99)
	JAK/STAT3	(99)
	Jun	(100, 101)
	HDAC	(102–105)
Stability or degradation	HER2	(106, 107)
	Na_2_S_4_	(30)
	HSPs	(108, 109)

### 3.1 Direct targeting of STAT3

The direct targeting approach to STAT3 involves specific interference with the protein’s structure or function to inhibit its activity, distinct from upstream pathway modulation. This strategy primarily focuses on five structural domains: the NTD, CCD, DBD, SH2 domain and TAD (56), which functionality was previously characterized. Recent studies have shown that direct targeting of STAT3 inhibits STAT3-mediated nuclear translocation, also induces STAT3 protein degradation via STAT3 inhibitors.

#### 3.1.1 Targeting NTD

Targeting the NTD directly acts on STAT3 by disrupting dimer function, DNA binding, and transcription complex assembly (57). The NTD mediates tetramerization of two Y705-phosphorylated STAT3 dimers, enabling cooperative binding to closely spaced STAT3 sites in gene promoters. Additionally, the NTD can also regulate Leukemia Inhibitory Factor (LIF) stimulation on the effect of STAT3 through numerous mechanisms, including chromatin remodeling, or the recruitment of transcriptional proteins, which influence the basal levels of STAT3 DNA binding or gene expression, partially regulating by cooperative STAT3 binding to tandem DNA motifs (58). ST3-H2A2, a selective inhibitor of NTD, inhibits STAT3 transcriptional activity by disrupting the binding between STAT3 dimers and DNA, inducing apoptotic death in cancer cells (59). However, the surface of the NTD is relatively flat, lacks deep pockets on the surface and represents a typical “non-druggable” protein, which makes the discovery of high-affinity inhibitors extremely challenging (60). Recent studies indicated that NTD has an important role when the activated STAT3 concentration is small (61).

#### 3.1.2 Targeting CCD

As a transformative binding domain of STAT3, the CCD promotes structural changes in the SH2 domain via helix–helix interactions, reducing SH2 binding affinity and consequently modulating STAT3 function (62). However, blocking the CCD can cause the structural rearrangement of STAT3 and hinder the dimerization of SH2 domain, which lead to STAT3 inactivation (63). MS3-6 (highly selective for STAT3) binds to the NTD and CCD domains, the crystal structure of STAT3 in complex with monobody MS3–6 reveals bending of CCD, resulting in diminished DNA binding and nuclear translocation (64). ASRPS directly bound to STAT3 through CCD and down-regulated STAT3 phosphorylation, which led to reduced expression of VEGF in breast cancer (65). K116, as a potent allosteric inhibitor specifically targeting CCD, inhibited the proliferation of breast cancer cell lines in a dose-dependent manner by reducing the phosphorylation of STAT3 Lyr705 (63).

#### 3.1.3 Blocking DBD

The DBD recognizes specific DNA sequences with high specificity and is linked to the SH2 domain via the linker domain, which regulate DNA binding and transcriptional activation of STAT3 (66). Notably, mutations in the DBD do not impair STAT3 functions downstream of pY-peptide binding, including Tyr705 phosphorylation, homodimerization, or nuclear accumulation (67). STAT3 inhibitors targeting DBD have been identified to have therapeutic potential in cancer, by suppressing the binding of dimerized STAT3 to target gene promoters through competitive inhibition. Targeting the DBD of STAT3, the inhibitor imatinib has exhibited potential to inhibit the growth, angiogenesis, and metastasis of colorectal cancer (68). Studies have shown that decoy oligodeoxynucleotides (dODNs) bind to the DBD of STAT3 dimers, which prevent STAT3 from binding to the promoter regions of endogenous target genes, resulting in transcriptional repression, reducing survival and tumor immune evasion in acute myeloid leukemia (69); Galiellalactone binds directly to the DBD of STAT3, thereby inhibiting its transcriptional activity, which in turn reduces the tumor growth, metastasis, and induces apoptosis of prostate cancer (70, 71).

#### 3.1.4 Targeting SH2 domain

Blocking the binding of monomeric STAT3 to phosphorylated tyrosine (pY705) via its SH2 domain, thereby suppressing SH2 domain dimerization and nuclear translocation, constitutes a core strategy for direct targeting of STAT3 (72). Compound 323–1 and 323–2 from the medicinal plant Incarvillea delavayi, named as (15R,2R)-delavatine A, directly targeting the SH2 domain and inhibited both phosphorylated and non-phosphorylated STAT3 dimerization, which are the promising lead compounds for the treatment of cancers with hyper-activated STAT3 (73). A novel small molecule inhibitor (N4) with potent anti-tumor activity could interact with the SH2 domain at multiple residues, including Lys591, Ser636, Val637, and Glu638, subsequently blocking STAT3 Tyr705 phosphorylation and significantly inhibiting pancreatic cancer growth and liver metastasis (74). YY002, a highly selective small-molecule STAT3 inhibitor, bounding to the SH2 domain, simultaneously inhibits both STAT3 Tyr705 and Ser727 phosphorylation. As a result, YY002 potently inhibited STAT3-dependent tumor cell growth *in vitro* and achieved potent suppression of tumor growth and metastasis *in vivo* (75). Benzethonium chloride (BZN) may directly bind to the SH2 domain of STAT3, inhibiting its dimerization, and preventing the nuclear translocation of phosphorylated STAT3. BZN not only significantly inhibited the proliferation and induced apoptosis of head and neck squamous cell carcinoma cell lines, but also markedly suppressed tumor growth in a subcutaneous tumor model (76). Moreover, SH2 domain inhibitors that block STAT3 dimerization effectively inhibit STAT3 nuclear translocation, DNA binding, and transcriptional activity. NSC74859 (targeting SH2) suppresses nuclear translocation of STAT3 dimers without altering their phosphorylation status, elicited potent antitumor immunity for enhanced cancer therapy (77, 78). Stattic is a small-molecule STAT3 inhibitor which selectively blocks the SH2 domain, thereby suppressing both STAT3 nuclear translocation and activation, and inducing cell death in T-cell acute lymphoblastic leukemia (79).

#### 3.1.5 Targeting TAD

The TAD, which is lacking in the predominant alternatively spliced isoform of STAT3, recruits co-activators with histone acetyltransferase activity to promote transcriptional activation. Ser727 within the TAD can be phosphorylated by multiple serine/threonine kinases, including mitogen-activated protein kinases (MAPKs), c-Jun N-terminal kinase (JNK), and protein kinase C (PKC) (80). It can translocate to mitochondrial membranes and interact with electron transport chain (ETC) components, affecting ROS production, tumor growth, and TME (81, 82). STAT3 inhibitors suppress transcriptional complex formation by disrupting interactions between STAT3 and coactivators such as p300/CBP. For instance, the inhibitor TAD-1822-7-F2 reduces STAT3 phosphorylation at both Tyr705 and Ser727, which shows an effective suppression for cervical carcinoma HeLa cells (83).

#### 3.1.6 Regulation of STAT3 negative feedback factors

The activity of STAT3 is negatively regulated by inhibitory proteins, including protein tyrosine phosphatases (PTPs), protein inhibitors of activated STATs (PIAS), and parts of suppressors of cytokine signaling (SOCS) (84). Upregulation of these factors can directly suppress STAT3 signaling. PTPs (including SHP1/2, PTPRD, PTPRT, PTPRK, PTPN9, and TC-PTP) inhibit STAT3 activation by decreasing STAT3 phosphorylation at Ser 727, negatively regulating cancer cells progression (85, 86). The overexpression of SHP1, which is closely associated with PTPN6 promoter methylation inhibits STAT3 activity in lymphoma and leukemia, thereby suppressing cancer progression (84). PIAS bind to STAT dimers and inhibit their DNA binding capacity, inducing SUMOylation of STATs, or enhancing the recruitment of other STAT co-repressors such as histone deacetylases (HDAC). Research has revealed that PIAS3 downregulated miR-21 in breast cancer by interfering with STAT3 DNA-binding activity (87). Experimental evidence demonstrates that SOCS protein activators inhibit STAT3 phosphorylation by binding to JAK or receptor complexes (88). Induction of methylation in CAFs suppresses SOCS1 expression and gene methylation, which enhances STAT3 phosphorylation and induces the expression of Insulin-like Growth Factor-1 (IGF-1), ultimately accelerating pancreatic ductal adenocarcinoma progression (89). SOCS proteins downregulate the upstream kinase activity responsible for STAT3 phosphorylation, while the PIAS proteins and PTPs target STAT proteins directly.

#### 3.1.7 Induction of STAT3 protein degradation

STAT3 inhibitors degrade STAT3 protein through either the ubiquitin-proteasome system (UPS) or lysosomal pathways. Experiments have demonstrated that bifunctional proteolysis-targeting chimera (PROTAC) molecules, such as SD-36, SD-91 and SD-2301, simultaneously engage STAT3 and recruit an E3 ubiquitin ligases, thereby inducing STAT3 for ubiquitination and degradation via the UPS (90–92). SD-2301 promotes efficient STAT3 degradation in lymphoma and impairs tumor cell proliferation via a high-affinity VHL-1 ligand and SH2-domain ligand. Simultaneously, SD-2301 fostered a favorable environment for CD8^4^ T cells by driving STAT3 depletion, reducing PD-L1 expression (93). Nanoengineered peptide-PROTACs degrade STAT3 effectively, leading to more effective antitumor effect in colorectal cancer (94). K63-linked polyubiquitination of STAT3 influences its phosphorylation and nuclear translocation, thereby modulating its transcriptional activity. Meanwhile, research demonstrates that USP20-medidate deubiquitination of STAT3 specifically removes K63-linked chains, which enhances STAT3 transcription and reduces its proteasomal degradation (95).

### 3.2 Indirect targeting of STAT3

Indirect targeting of STAT3 suppresses its function by inhibiting upstream activating signals, modulating associated regulators, or compromising its stability. Compared with direct STAT3 targeting, this strategy mitigates toxicity and may overcome drug resistance.

#### 3.2.1 Inhibition of upstream signaling pathway activation

The phosphorylation and activation of STAT3 are highly dependent on upstream signaling molecules, including JAK kinases, cytokine receptors, and growth factor receptors. Notably, key ligands such as IL-6, IL-23R, platelet-derived growth factor (PDGF), fibroblast growth factor (FGF), epidermal growth factor (EGF) are the most potent activators. Inhibiting these pathways can indirectly block STAT3 activity (96, 97). The data indicated that the targeting of IL-6/JAK2/STAT3 signaling in activated microglia may be a promising new approach for inhibiting brain metastasis in non-small cell lung cancer patients (98).

#### 3.2.2 Inhibition of STAT3 co-regulators

The function of STAT3 relies on coactivators (such as NF-κB and Jun) or epigenetic modifying enzymes (e.g., HDAC). Activation of NF-κB released proinflammatory factors that facilitated STAT3 phosphorylation and increased tumor cell proliferation and invasion. Dimethyl fumarate inducing ferroptosis and then having a broad antitumor effect was mainly characterized by inhibition of NF-κB and JAK/STAT3 signaling in diffuse large B-cell lymphoma (99). As a co-regulator of STAT3, Jun depletion in a Pten-deficient background has been shown to prevent immune cell attraction, which was accompanied by significant reduction of active STAT3 and accelerated prostate cancer growth (100). In melanoma cells, STAT3 oncogenic activities could be mediated through its cooperation with Jun, resulting in downregulation of Fas surface expression, which is implicated in the tumor’s ability to resist therapy and metastasize (101). HDAC is usually aberrantly activated in tumors. HDAC6, a key histone deacetylase in the HDAC family, intervenes the presence of STAT3 acetylation (K685), leaving the SH2 domain of STAT3 free (102). Additionally, HDAC6, which physically interacts with STAT3, regulates STAT3 phosphorylation (Y705), and the expression of prostaglandin E2/cyclooxygenase-2 (COX2), programming the immunosuppressive tumor microenvironment (103, 104). Thus, the approved pan-HDAC inhibitors (HDACi) have exhibited clinical benefits for anticancer therapy. The combination of regorafenib and JAK/HDACi not only decreased STAT3 phosphorylation at Y705 but also inhibited deacetylation at K685, leading to enhanced the antitumor immune response in colorectal cancer (105).

#### 3.2.3 Indirect impact on STAT3 stability or degradation

The stability and degradation of STAT3 are primarily indirectly regulated by the phosphatases, ubiquitin-proteasome system, molecular chaperones. By targeting these upstream regulators rather instead of STAT3 directly, it is possible to modulate STAT3 stability or degradation in cancer. Human Epidermal Growth Factor Receptor 2 (HER2) activation resulted in phosphorylation of STAT3, which leads to increased ubiquitination and accelerated degradation (106, 107). In renal tubular epithelial cells, Na_2_S_4_ induced dephosphorylation and deacetylation of STAT3, thereby reducing high glucose-caused oxidative stress, cell apoptosis, inflammation response and epithelial-to-mesenchymal transition progression (30). Ubiquitin Specific Peptidase 5 (USP5) stabilized STAT3 via its deubiquitination function, thereby enhancing the production of pro-angiogenic factors by cancer cells, driving tumor angiogenesis (16). Heat shock proteins (HSPs) are molecular chaperones, binding to STAT3 and protecting it from ubiquitination and proteasomal degradation, thereby maintaining STAT3 stability. HSPs, including HSP70, HSP90, HSP110 and HSP27 have represents promising therapeutic targets in myeloproliferative neoplasms (108, 109).

## 4 Combination of STAT3 and immunotherapy

**Figure 4:**
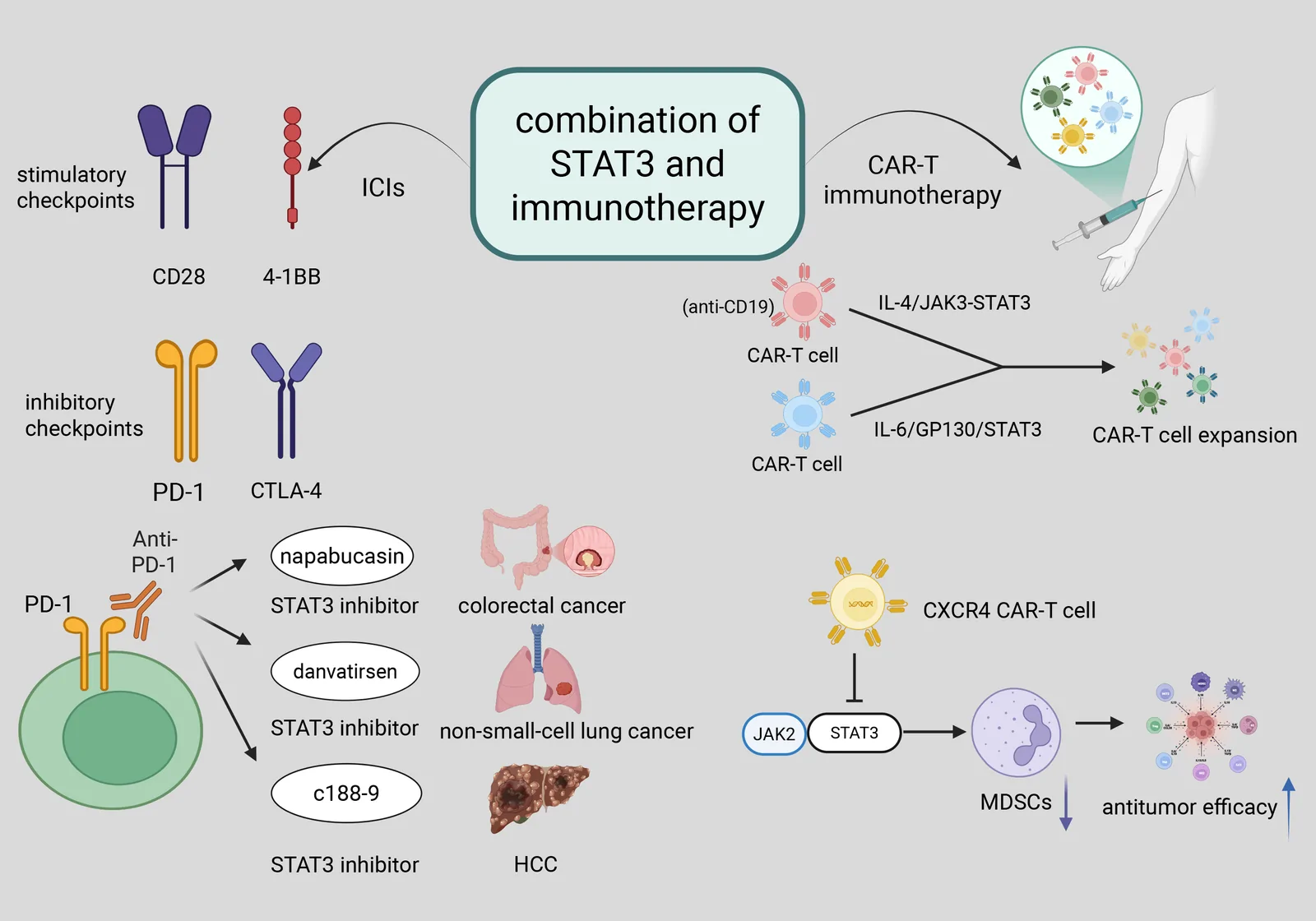
Combination of STAT3 and immunotherapy. Major immunotherapeutic approaches include ICIs and CAR-T immunotherapy. The left part of the picture demonstrates combined STAT3 inhibition with ICIs, including stimulatory checkpoints, inhibitory checkpoints and the combination examples in different kinds of cancer models. The right part demonstrates the involvement of STAT3 signaling and inhibitors in CAR-T therapy. ICIs, immune checkpoint inhibitors; CAR-T, chimeric antigen receptor T-cell. Created with https://www.Biorender.com.

Cancer immunotherapy represents a therapeutic strategy that activates or enhances the patient’s own immune system to recognize, attack, and eliminate cancer cells. Unlike conventional treatments (surgery, chemotherapy, and radiotherapy), its core principle lies in leveraging the immune system’s ‘memory’ and ‘specificity’ to achieve long-term antitumor effects. Major immunotherapeutic approaches include immune checkpoint inhibitors (ICIs) and chimeric antigen receptor T-cell (CAR-T) immunotherapy (**Figure 4**).

### 4.1 Combination of STAT3 inhibition with ICIs

Immune checkpoints are key regulatory molecules that modulate immune responses, including stimulatory checkpoints (e.g., CD28, 4-1BB) and inhibitory checkpoints (e.g., PD-1/PD-L1, CTLA-4). ICIs restore T cell-mediated antitumor activity by blocking inhibitory signals or enhancing stimulatory signals (110). However, aberrant STAT3 activation substantially diminishes ICI efficacy. Combined STAT3 inhibition with ICIs synergistically reverses immunosuppression and enhances therapeutic outcomes. The putative promoter region of the PD-L1 gene, located upstream of the 5′ end, contains four potential STAT3 binding sites (111). The activation of STAT3 binds to the PD-L1 promoter, upregulating the interaction with PD-1 on CD8^+^ T cells, forming an immunosuppressive microenvironment and facilitating tumor growth (112). Studies have demonstrated that STAT3 inhibitors can block STAT3 phosphorylation and reduce PD-L1 expression in both tumor cells and immune cells. When combined with PD-1/PD-L1 inhibitors, this approach effectively alleviates T cell inhibitory signals and enhances cytotoxic activity. Although, the STAT3 inhibitor napabucasin has been studied in combination with anti-PD-1 inhibitor in patients with refractory or intolerant metastatic colorectal cancer, its hypothesized clinical benefit has not been observed up to date (113). Danvatirsen, a STAT3 inhibitor, combining with immune checkpoint inhibitors, is currently being tested in a phase 2 umbrella trial for advanced non-small-cell lung cancer. However, its hypothesized clinical benefit has not been observed up to date (114). C188-9 (the highly selective STAT3 inhibitor) has progressed to phase I clinical trials in combination with PD-1 antibody therapy for advanced hepatocellular carcinoma (2, 115). The promising early phase clinical trials encourage further clinical development of this combination strategy.

### 4.2 Combination of STAT3 modulation with CAR-T cell therapy

CAR-T cell therapy has emerged as a cancer treatment with enormous potential, especially for hematological malignancies (116). The involvement of STAT3 signaling in CAR-T therapy is emerging. The study showed that anti-CD19 CAR-T cells from responsive patients with chronic lymphocytic leukemia had an increased IL-4/JAK3-STAT3 signaling pathway, which promoted the expansion of CAR-T cells (117). Studies also have shown that IL-6 trans-signaling can enhance expansion and antitumor activity of CAR-T cells via the IL-6/GP130/STAT3 pathway (118). These studies suggest a beneficial effect of STAT3 activation in CAR-T cells.

As mentioned above, STAT3 in TAM can increase the induction of immune evasion. Thus, there are some attempts to combine CAR-T therapy with STAT3 inhibition to improve the persistence and anti-tumor effects, as well as negating toxicities of CAR-T cells *in vivo*. The JAK2/STAT3 axis is a crucial driver of liver-associated MDSCs. Researchers suggest that inhibiting STAT3 increased the efficacy of CAR-T cells in liver cancer metastasis (72). CXCR4-modified CAR-T cells suppress STAT3 activation within CAR-T cells, reduce the recruitment of MDSCs to TME, and enhance antitumor efficacy in pancreatic cancer (119).

## 5 Conclusion and future perspectives

STAT3 is activated in diverse human malignancies and serves as a pivotal signaling hub in both tumor cells and components of TME, particularly tumor-infiltrating immune cells. This central role position makes STAT3 as a promising therapeutic target in oncology. Therapeutic strategies include direct or indirect suppression of STAT3 functional domains. Notably, ongoing combination therapies with immune checkpoint inhibitors have expanded response rates and the spectrum of treatable cancers. The synergy between STAT3 inhibitors and conventional antitumor agents (including immunotherapies) may enable durable, multi-layered tumor control. While preclinical and early-phase clinical trials of dual STAT3/checkpoint inhibition show encouraging results, their translational potential warrants further validation. Collectively, targeting STAT3 signaling demonstrates significant promise for restoring antitumor immunity and represents a compelling avenue for advancing cancer immunotherapy.
